# Springback Analysis and Prediction of Automotive Steel Sheets Used in Compression Bending

**DOI:** 10.3390/ma18040774

**Published:** 2025-02-10

**Authors:** Emil Spišák, Janka Majerníková, Peter Mulidrán, Július Hajduk, František Ruda

**Affiliations:** 1 Institute of Technology and Materials Engineering, Faculty of Mechanical Engineering, Technical University of Košice, Mäsiarska 74, 040 01 Kosice, Slovakia; janka.majernikova@tuke.sk (J.M.); peter.mulidran@tuke.sk (P.M.); julius.hajduk@student.tuke.sk (J.H.); 2 U. S. Steel Košice, s. r. o., Vstupný Areál U. S. Steel, 044 54 Kosice, Slovakia; fruda@sk.uss.com

**Keywords:** springback, springback prediction, TRIP, micro-alloyed steel, anisotropy, compression bending

## Abstract

Springback is still one of the most challenging issues in sheet metal forming, especially in the automotive industry, where hundreds of pressings made of different types of materials are joined together by various methods. In this study, springback evaluation was conducted on three types of steel that are commonly used in the automotive industry. Transformation induced plasticity (TRIP), micro-alloyed and deep-drawing steel sheets were used in compression bending, which is largely used in bending hollow tubes. The impact of die diameter and anisotropy on the springback was studied and evaluated. A numerical prediction of springback was conducted using Simufact Forming 2022 software. Based on the experimental results, it was found that steels with higher yield and tensile strength experience more springback than steels with lower strength properties. Additionally, the use of larger die diameters (30 mm and 25.4 mm) in compression bending results in higher springback compared to smaller ones (10 mm and 15 mm). The impact of anisotropy on the springback was mostly evident in pressings made of deep-drawing steel.

## 1. Introduction

Springback, a common post-forming phenomenon, arises from elastic recovery after external forces are removed. This can result in dimensional inaccuracies and assembly difficulties [1,2,3]. Springback poses significant challenges to automotive manufacturing, in which car bodies are composed of numerous sheet metal components with varying mechanical properties. These components, that are assembled through various techniques including spot welding, clinching, and gluing, often require precise dimensional accuracy, which can be compromised by springback [4,5,6]. The springback is higher in high-strength steels compared to conventional steels, as these undergo a larger proportion of elastic deformation during forming [7]. High-strength steels, that are often employed in critical structural components such as A and B pillars, front rails, and door/roof reinforcements, are susceptible to springback, potentially impacting the accuracy of parts and their assembly process [8,9,10].

Micro-alloyed and transformation induced plasticity (TRIP) steels are among the most commonly used high-strength steels in the automotive industry. Micro-alloyed steel sheets are a type of steel that has been enhanced with small additions of elements such as niobium, vanadium, titanium, and nitrogen. These additions, known as microalloying elements, significantly improve the mechanical properties of the steel, such as strength, toughness, and formability [11,12]. The microstructure of micro-alloyed steels is characterized by fine-grained ferrite and pearlite phases. The microalloying elements promote the formation of fine precipitates, which impede dislocation movement and strengthen the steel [13]. The fine-grained microstructure of these steels enhances their formability, making them suitable for complex forming processes in the automotive industry. Micro-alloyed steels also exhibit good weldability, ensuring the reliable joining of components [11].

TRIP steel sheets are a class of advanced high-strength steels (AHSS) known for their exceptional mechanical properties [14]. Their microstructure comprises multiple phases: ferrite, a ductile phase enhancing formability, retained austenite, a metastable phase transforming into martensite during deformation for increased strength, and bainite, a hard phase contributing to overall strength [15]. TRIP steels offer a superior strength-to-weight ratio, excellent formability, and enhanced energy absorption. These properties make them ideal for various automotive applications, including safety-critical components such as B-pillars and side sills, as well as body in white components including door reinforcements and roof panels [16,17].

Most of the existing research aimed at springback prediction and analysis of steel sheets has used some type of bending process. The majority of publications regarding springback analysis used some type of V or U bending process [18,19,20]. Researchers have often studied the impacts of material properties, process parameters and tool geometry on the springback effect [21]. The finite element method (FEM) is often used to predict springback of sheet metal parts produced by forming [22]. To ensure the accuracy and reliability of numerical simulations in metal forming, it is crucial to input correct parameters and conditions. Four primary categories of parameters significantly impact simulation accuracy: tool design, process conditions, material properties, and numerical settings [20,23]. Slota and Jurčišin [24] investigated the deformation behavior of TRIP, AHSS, and mild steel during a V bending process using finite element analysis (FEA). Based on their simulations, they developed a predictive model to estimate springback in these materials during the air bending process. The work of Wasif et al. [25] aimed to study the impact of thickness, width, and bend angle of two types of high-strength steels produced by the V-bending process. These authors used analysis of variance to study the magnitude of influence of these parameters on the springback. Their results suggest that thickness of steel sheet and bend angle have significant impact on the springback. A hat-shaped part produced by U bending was used by Yoshida-Uemori [26] for verification of their proposed hardening model, which can describe the transient Bauschinger effect and work hardening stagnation, both of which appear at reverse loading. The implementation of the Bauschinger effect and work hardening stagnation can improve springback prediction [20,21,22]. This shape is also present in reinforcement beams of doors and roofs of passenger cars that are made of materials with high yield and tensile strength values. Konzack et al. [18] studied the impact of blank holding force on the springback prediction of a hat-shaped part made of AHSS steel. They found out that the Hill48 yield criterion was more accurate in predicting springback when a higher value of blank holding force was applied during forming, compared to the Yoshida yield criterion. Mulidrán et al. [27] used a hat-shaped part made of dual-phase steel to study the impact of friction and blank holding force on the springback of the produced part. In this study, different combinations of yield criteria and hardening models were used to study their impact on the accuracy of springback predictions. Their results suggest that use of PE foil can significantly reduce springback.

Steel stock is often processed by bending to produce desired parts. The most common sheet metal bending operations include U bending, V bending, rotary bending, edge bending, draw bending, and air bending [28]. The current study details the springback evaluation and prediction of sheet metal parts produced by compression bending. The effects of die diameter and anisotropy on the springback were studied in three types of steels—TRIP, micro-alloyed and deep-drawing steel—that are commonly used in automotive production. The novelty of this work is its comprehensive analysis of the impacts of die radius and anisotropy on the springback of sheet metal parts produced by compression bending, which is predominantly used for bending hollow tubes. Furthermore, a numerical analysis was conducted, in order to study the impact of yield criteria and element size on the accuracy of springback prediction.

## 2. Materials and Methods

### 2.1. Materials

Three types of steel commonly used in the automotive industry were used in the present work. Two high-strength steels—0.70 mm thick micro-alloyed HX420 (material A) and TRIP steel RAK 40/70 (material B) with a thickness of 0.75 mm and one extra deep-drawing quality steel DC06 (material C) with a thickness of 0.85 mm—were used in the experiment. Steels exhibit distinct deformation behaviors due to their unique microstructures [29,30,31]. Understanding these material properties and characteristics is crucial for accurate numerical simulations of forming processes [31,32]. The chemical composition of the tested steels was determined using a Belec Compact Port spectrometer (Belec Spektrometrie Opto-Elektronik GmbH, Georgsmarienhütte, Germany), and the chemical compositions are presented in Table 1.

**Figure 1:**
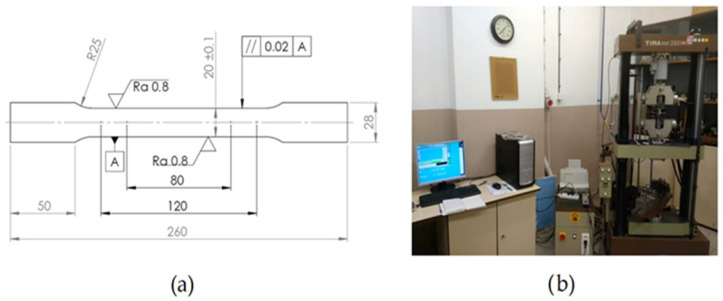
Dimensions of tensile test specimen (**a**); universal testing machine TIRAtest2300 (**b**).

Uniaxial tensile tests on standardized specimens (Figure 1a) were conducted to determine the mechanical and plastic properties of the steels used in this study. The tests were performed according to the STN EN ISO 10113: 2020 [33], STN EN ISO 10275: 2020 [34], and STN EN ISO 6892-1:2019 [35] standards on a TIRAtest 2300 device (Figure 1b).

To measure the values of the mechanical properties of the investigated materials, five samples were produced and tested from each material in the directions of 0°, 45° and 90° with respect to the rolling direction. The obtained values of the tested materials A, B and C according to the results of uniaxial tensile tests are shown in Table 2, Table 3, and Table 4, respectively.

### 2.2. Experimental Setup

**Figure 2:**
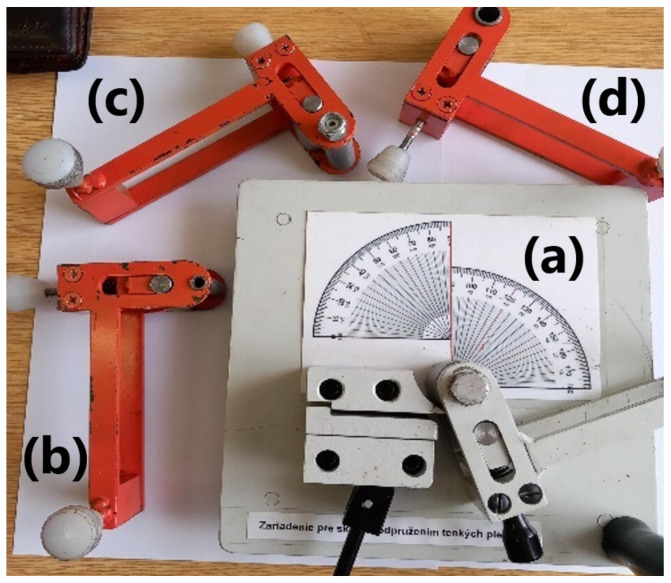
Compression bending tool (**a**) with different sets of bending dies displayed: (**b**) 10 mm set, (**c**) 15 mm set, and (**d**) 20 mm set.

A compression bending tool (Figure 2) was designed to study springback in three types of steels that are frequently used in the automotive industry. A similar tool design is used in the so-called “Springback test” to evaluate tin plates’ hardness and yield strength [36]. In compression bending, the material is deformed gradually along the circumference of the fixed cylindrical die. Our tool enables changes in the diameter of moving and stationary die, thus varying stress and strain during bending of sheet metal. The tool is manually operated; the moving die is worked by human power using the handle.

**Figure 3:**
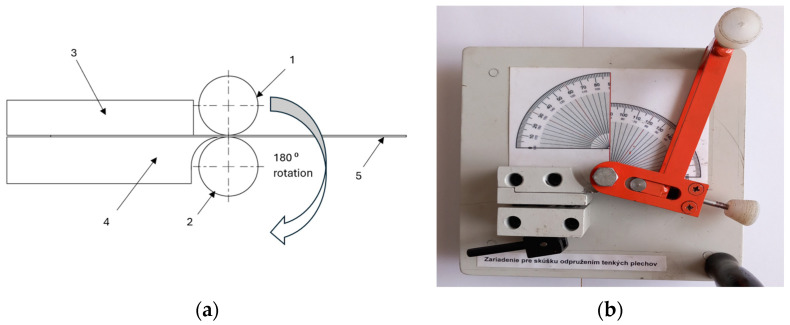
Schematics of the compression bending tool (**a**): 1. movable die; 2. fixed die; 3. blank holder; 4. base plate; and 5. sheet metal blank; compression bending tool (**b**).

The experimental procedure comprised the compression bending of the material using a movable and fixed die. Our tests used sheet metal samples measuring 152.4 × 25.4 mm, which were clamped into the tool at one end and the other free end was gradually bent to 180° around the cylindrical surface of a fixed mandrel using a moving mandrel of the same diameter (Figure 3). To study the effect of the die diameter on the amount of springback, five sets of bending dies (fixed and movable dies with same diameter) were used—30.0 mm, 20.0 mm, 25.4 mm, 15.0 mm, and 10.0 mm (Figure 2). The 25.4 mm diameter dies are used in the previously mentioned “Springback test”. Materials A, B and C were used in the bending experiments. Five samples were made and tested from each type of material in directions of 0°, 45° and 90° with respect to the rolling direction in five sets of bending dies. Overall, seventy-five samples were tested in the compression bending tool. This was carried out to study the effects of die diameter and anisotropy on the springback of bent steel sheets.

### 2.3. Simulation Setup

**Figure 4:**
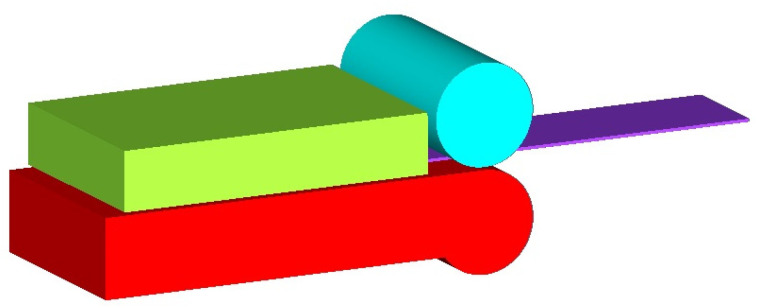
CAD model of the compression bending tool used in the numerical simulation.

The springback simulations were performed using Simufact Forming 2022 software (Hexagon AB, Stockholm, Sweden). This software is widely used for sheet metal and bulk forming simulations. The CAD model of the bending tool with a 25.4 mm die diameter (Figure 4) was imported into the software, and blank dimensions and its position were defined. The blank was mashed with shell elements, and the number of elements through thickness ranged from 2, 4, 6, up to 8 elements, thus, affecting the size and number of elements in the sheet metal blank. The material properties of HX420, RAK 40/70, and DC06 steels were defined using two different yield criteria and one isotropic hardening law. To represent bending without lubrication, the coefficient of friction (COF) was set to 0.20. The COF value was selected based on studies of Evin and Trzepiecinski [37,38]. The simulations were designed to analyze the changes in predicted springback caused by using different material models and different sizes of elements. Numerical springback results were compared with experimental ones.

#### Yield Criteria and Hardening Model

Cold-rolled steel sheets exhibit anisotropic plastic behavior due to their manufacturing process. This anisotropy manifests in differences in mechanical properties along the rolling, transverse, and thickness directions. Furthermore, deformation-induced anisotropy, arising from microstructural changes during forming, further complicates the material’s response. To accurately simulate forming processes, it is essential to model the material’s anisotropic yielding behavior [39,40,41]. Hardening models, sometimes recognized as hardening laws or rules, describe how the yield surface transforms during the plastic deformation of sheet metal [42]. Three main types of hardening rules, isotropic, kinematic, and isotropic–kinematic, can be used to describe the hardening of metallic materials in forming simulations [42].

To define elasto-plastic behavior of sheet metal in numerical simulations, it is necessary to input data from various tests, mainly uniaxial tensile tests. These data are used for defying the yield criteria and hardening models. In this study, two yield criteria, Hill48 [43] and Barlat91 [44], were used to describe yielding behavior of the material. To describe changes of the yield surface, the Hollomon isotropic hardening model (Table 5) was used. Parameters for definition of yield criteria are displayed in Table 6. The isotropic hardening model was defined as:

(1) $$\sigma =K\cdot \varphi^{n}$$

where σ defines the true stress, K is the strength coefficient, n represents the strain-hardening exponent, and φ defines the plastic strain.

## 3. Results

The springback angles were measured and evaluated for three types of steels used in the experiment. Measured springback angles were in the range of 6° to 58°. The impacts of anisotropy and die diameter on the springback were evaluated for each tested steel. The springback angle α [°] from experimental measurement of samples produced from sheets cut in 0° to the rolling direction in a tool with 25.4 mm diameter dies was compared with the angle from numerical simulations. In simulations, the impacts of the used material models and element size on the accuracy of springback prediction was studied and investigated.

### 3.1. Analysis of Springback Results from Experiment

**Figure 5:**
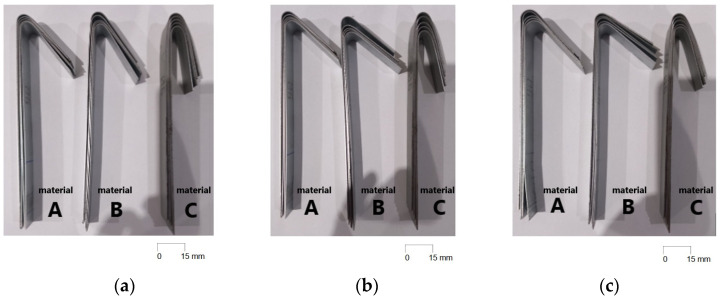

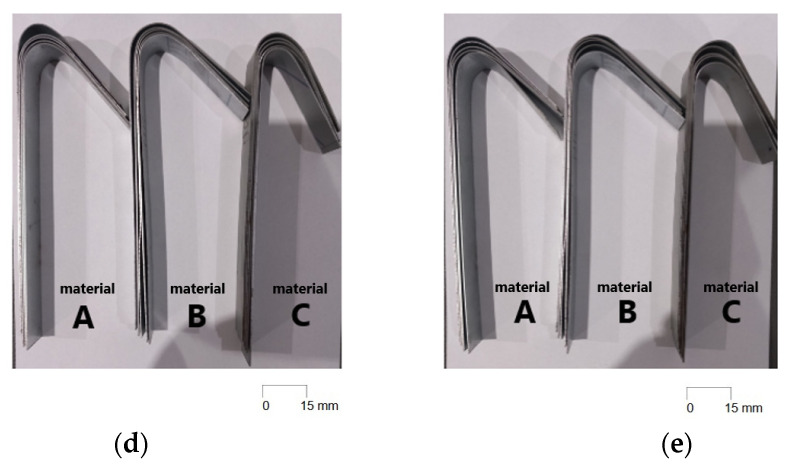
Materials A, B and C formed in the compression bending tool with different die diameters: (**a**) 10 mm die diameter; (**b**) 15 mm die diameter; (**c**) 20 mm die diameter; (**d**) 25.4 mm die diameter; (**e**) 30 mm die diameter.

The impacts of die diameter and anisotropy on springback were tested and examined on three types of steels used in the automotive industry. Five sets of die diameters were tested for each type of material. The impact of anisotropy was tested by cutting the blank at 0°, 45° and 90° with respect to the rolling direction. In total, seventy-five samples were produced using the compression bending tool. Figure 5 shows samples made of HX420, RAK 40/70, and DC06 steel after compression bending using different die diameters.

**Figure 6:**
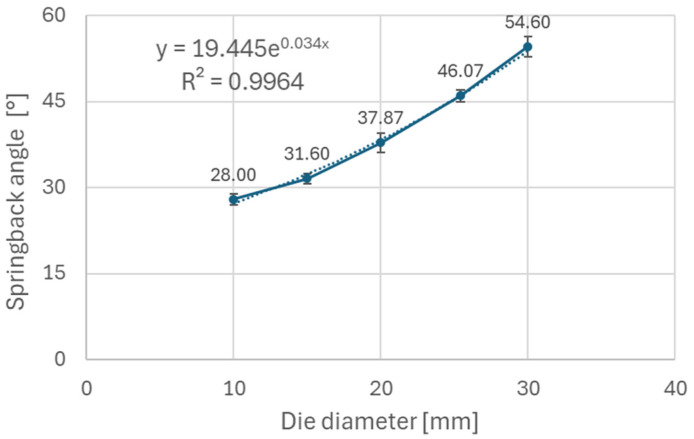
Dependence of springback angle on die diameter for material A.

**Figure 7:**
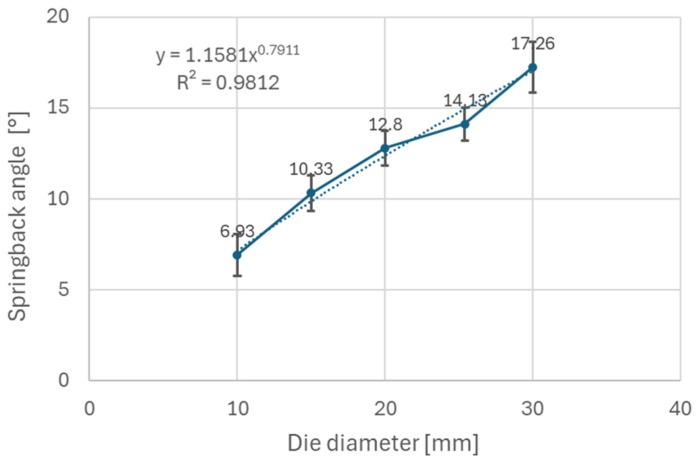
Dependence of springback angle on die diameter for material B.

**Figure 8:**
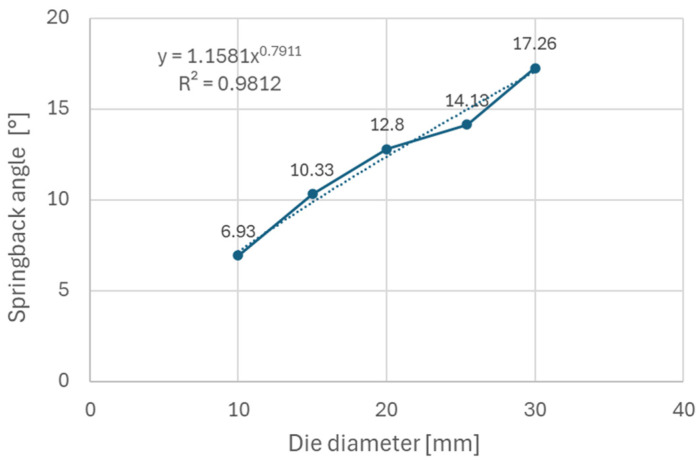
Dependence of springback angle on die diameter for material C.

It can be observed (Figure 5) that steels with higher yield strength (materials A and B) have a more prominent springback effect compared to the low strength DC06 steel (material C). Additionally, the use of larger die diameters (e.g., 30 mm diameter) resulted in a stronger springback effect compared to smaller die diameters (e.g., 10 mm diameter). This can be attributed to lower plastic deformation when larger diameters of dies are used in the bending, thus the impact of elastic deformation is more noticeable after forming. Figure 6, Figure 7, and Figure 8 show dependence between the die diameter and springback angle (average values of obtained springback from samples cut at 0°, 45° and 90° with respect to the rolling direction) of materials A, B and C, respectively. The smallest values of springback angle were measured in samples that were produced in the bending tool with 10 mm diameter dies. The average values of springback angle for DC06, HX420, and RAK 40/70 after bending with 10 mm dies were 6.93°, 28.0°, and 30.6°, respectively. The largest values of average springback angle were measured when 30 mm dies were used in bending. The DC06 steel springback was in the region of 17.26°, with HX420 at 54.60°, and the RAK 40/70 average springback angle value was 58.60° when 30 mm dies were used in compression bending.

**Figure 9:**
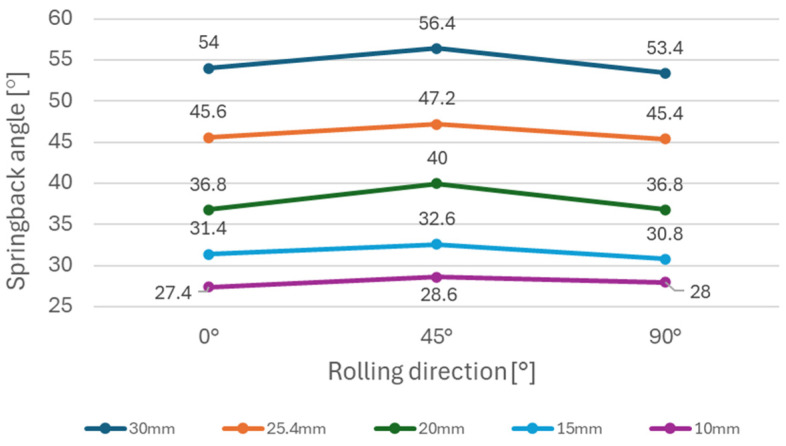
Impact of rolling direction on springback using different die diameters for material A.

**Figure 10:**
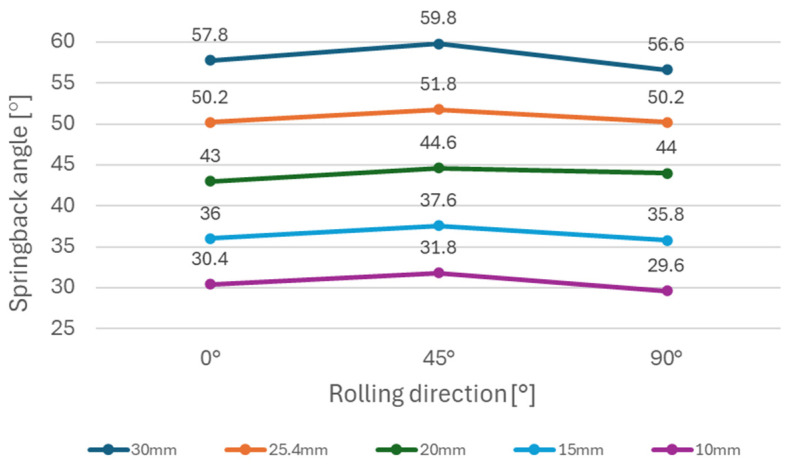
Impact of rolling direction on springback using different die diameters for material B.

**Figure 11:**
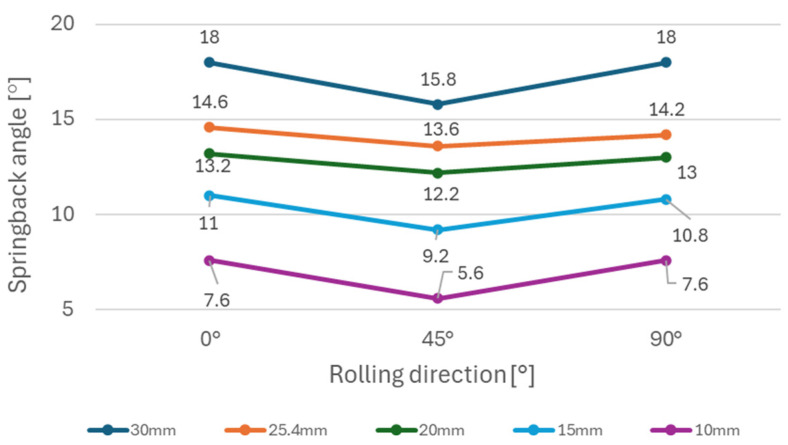
Impact of rolling direction on springback using different die diameters for material C.

Figure 9 shows the dependence of the springback angle on the material rolling direction for the HX420 (Material A) tested on different bending die diameters. This material, similarly to material TRIP RAK 40/70 (as shown in Figure 10) experienced more springback in 0° and 90° directions in comparison with the 45° direction. The impact of anisotropy is more visible in general when larger die diameters are used. Figure 10 shows the impact of anisotropy on the springback of TRIP steel (Material B). This material experienced the strongest springback effect of all tested materials. Material C—DC06 steel (Figure 11) —compared to other two steels, had the lowest values of springback angle after bending. In the case of DC06 steel, the lowest springback angle was measured in the 45° direction.

Based on the experimental results, it can be stated that materials with higher values of yield strength (Materials A and B) experience more springback after compression bending compared to material with a lower value of yield strength (Material C). The impacts of die radius and anisotropy were studied on the above mentioned materials. Die radius impacts the springback much more than does anisotropy of the material. For example, the RAK 40/70 steel springback angle value ranged from 30.4° to 57.8° when using different diameters of die. The difference of springback angle when samples are cut and tested in different directions is minimal; in the case of RAK 40/70 steel, this difference is around 2°.

### 3.2. Analysis of Springback Results from Simulation

**Figure 12:**
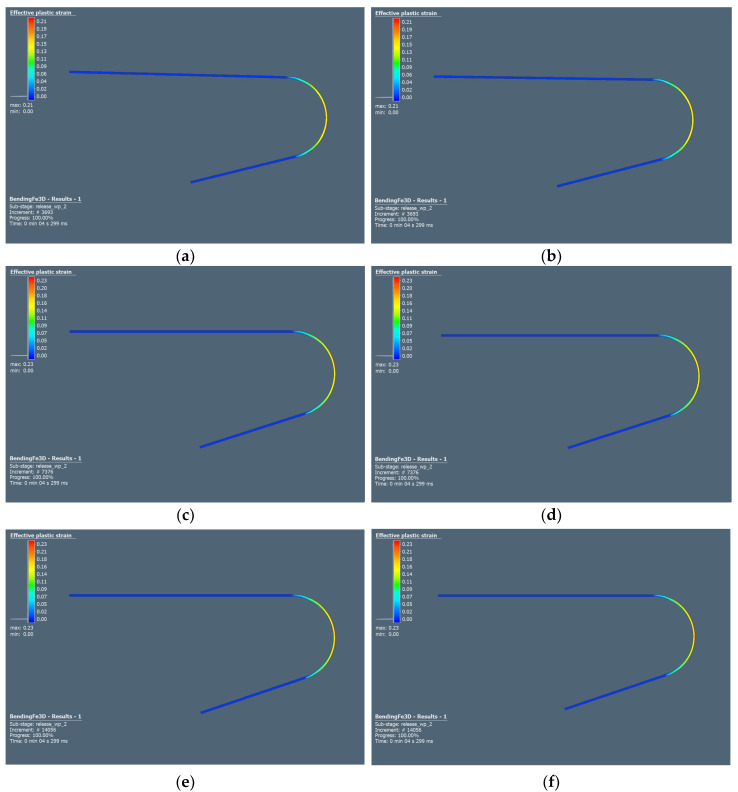
Springback prediction of TRIP RAK 40/70 steel using different size of elements and material models (**a**) Barlat91 yield criterion and element size of 0.37 mm; (**b**) Hill48 yield criterion and element size of 0.37 mm; (**c**) Barlat91 yield criterion and element size of 0.185 mm; (**d**) Hill48 yield criterion and element size of 0.185 mm; (**e**) Barlat91 yield criterion and element size of 0.0925 mm; (**f**) Hill48 yield criterion and element size of 0.0925 mm.

The impact of yield criteria and element size on the springback prediction accuracy was studied. Hill48 and Barlat91 yield criteria were used in combination with the Hollomon isotropic hardening law. Figure 12 shows the RAK 40/70 steel bent sheet metal after forming and springback simulations, where different sizes of elements were tested. This simulation was performed using the above mentioned yield surface models in combination with the Hollomon hardening law.

From Figure 12, only a slight change of arm angle after springback is noticeable when different yield criteria are used. The change in element size from 0.37 to 0.0925 had an impact on the predicted springback angle; for example, a difference of around 3° was recorded in the case of simulations where Barlat91 was used.

Based on the springback prediction results and their deviations from measured springback angles, it can be stated that numerical simulations were not able to accurately predict the impact of elastic deformation and springback for materials A and B (Table 7 and Table 8). Deviations between measured and predicted springback angles were in the range of 31.45° to 35.75° for material A. Similar deviations can be observed in material B. The predicted springback angle for material C (Table 9) shows a more accurate estimate of the impact of elastic deformation on the overall shape of the formed sheet metal. The difference between measured and predicted angles ranged from 2.7 to 6.0 degrees. In the case of DC06 steel, the smallest deviations were measured when the Hill48 yield criterion was used in the simulation. The size of the element used influenced the springback prediction. The smaller the size of the element used, the more accurate were the prediction results.

## 4. Discussion

This study evaluated the influences of die diameter and material anisotropy on the springback effect in the compression bending process, in which three types of steels used in the automotive industry were tested. The compression bending process of sheet metal is not typically employed in industrial practice. Findings presented in this article can be applied in industrial and automotive practice, where compression bending process of thin sheets is used. Primarily, the results regarding the impacts of die diameter and sheet metal anisotropy on springback can be useful when dealing with the springback phenomenon. Recent research has primarily focused on the impact of material and process parameters on springback prediction accuracy in numerical simulations [20,21,26]. The current work also aimed to investigate the roles of material models and element size in springback prediction.

The experimental results (Figure 6, Figure 7 and Figure 8) show that steels with higher yield strengths, such as materials HX420 (Material A) and RAK 40/70 (Material B), exhibited more pronounced springback compared to the lower strength DC06 steel (material C). Similar findings regarding the impact of strength characteristics were published by Jurcisin [45] and Mulidran [6]. Additionally, larger die diameters resulted in increased springback due to reduced plastic deformation and a greater influence of elastic recovery. The least springback was observed in samples bent using 10 mm diameter dies. For DC06, HX420, and RAK 40/70 steels, the average springback angles were 6.93°, 28.0°, and 30.6°, respectively, when bent with 10 mm dies. On the contrary, the maximum average springback angles were recorded when 30 mm dies were used: 17.26° for DC06, 54.60° for HX420, and 58.60° for RAK 40/70. Similar findings regarding the impact of die and punch radius on the springback can be found in the works of Buang et al. [46] and Jurcisin [45]. The impact of material anisotropy on springback was less prominent compared to the impact of die diameter (Figure 9, Figure 10 and Figure 11). Samples of DC06 steel prepared in a 45° direction had up to 13% less springback after bending compared to samples prepared in 0° and 90° directions (Figure 11).

The numerical results show that the springback was underestimated in all predictions and thus the impact of elastic deformation on the total deformation was underrated. Numerical simulations were not able to accurately predict the impact of elastic deformation and springback for the high-strength materials HX420 and RAK 40/70 steel (Table 7 and Table 8). The main reason for prediction inaccuracy might be the use of the Hollomon isotropic hardening rule, which cannot predict the Bauschinger effect and workhardening stagnation after reverse loading [26,27].

The predicted springback angle of material C (low strength DC06 steel) shows a more accurate estimate of the impact of elastic deformation on the overall shape of the formed sheet metal. The difference between the measured and predicted angles ranged from 2.7 to 6.0 degrees (Table 9). The size of the element used influenced the springback prediction. The smaller the size of the element used, the more accurate the prediction results achieved, which is in accordance with the works of Wagoner [47] and Liu [48]. They [47,48] also suggest that 8–11 integrations points through the thickness are sufficient for accurate springback prediction. Chatti [49] performed an investigation in which he studied the effect of the number of elements through thickness on the predicted springback angle; his results suggest that eight elements are sufficient for accurate prediction.

## 5. Conclusions

Springback is still a huge challenge that is present in sheet metal forming processes. In this study, the impact of die diameter (ranging from 10 mm up to 30 mm) and material anisotropy on the springback of three automotive steels, DC06, HX420, and RAK 40/70, in compression bending was studied. To evaluate springback prediction accuracy, two different yield criteria were used in combination with the Hollomon hardening rule in Simufact Forming software. Based on the experimental and numerical results, the following conclusions can be stated:

- Die diameter has a significant impact on the springback of the tested steels, and use of smaller die diameters decreases the impact of elastic deformation on total deformation, thus reducing springback. Parts made in a tool with 10 mm die diameter had approximately 50% less springback than parts made in a tool with 30 mm die diameter.
- The impact of material anisotropy on springback was less prominent compared to the impact of die diameter. Anisotropy had the greatest effect on the springback angle when DC06 steel was used in the experiment. Samples of DC06 steel prepared in the 45° direction had up to 13% less springback after bending compared to samples prepared in the 0° and 90° directions.
- The Hollomon hardening rule in combination with Hill48 and Barlat91 yield criteria demonstrated inaccurate springback predictions when the high strength steels RAK 40/70 and HX420 were tested in a simulation of the compression bending process. Accuracy of springback predictions was more satisfactory when DC06 steel was used in the simulation.
- Springback predictions were closer to experimental values when a smaller size of elements was employed in simulations.

## Figures and Tables

**Table 1:** Chemical composition of experimental materials (weight %).

Material	C	Mn	Si	Cr	P	S	Al	Nb	Ti
A	0.05	0.71	0.26	0.05	<0.002	<0.002	0.03	0.032	<0.002
B	0.2	1.27	0.14	0.04	0.019	<0.002	2.56	0.016	0.005
C	0.002	0.06	0.02	0.02	<0.002	<0.002	0.04	0.006	0.006

**Table 2:** Mechanical properties and formability parameters of HX420 steel (material A).

Dir. [°]	R_p0.2_ [MPa]	R_m_ [MPa]	A_80_ [%]	*r* [-]	*r_m_* [-]	*n* [-]	*n_m_* [-]
0	468	522	22.2	0.790		0.143	
45	465	512	24.0	1.096	0.895	0.142	0.144
90	454	522	18.8	0.599		0.151	

**Table 3:** Mechanical properties and formability parameters of RAK 40/70 steel (material B).

Dir. [°]	R_p0.2_ [MPa]	R_m_ [MPa]	A_80_ [%]	*r* [-]	*r_m_* [-]	*n* [-]	*n_m_* [-]
0	435	765	29.6	0.702		0.298	
45	443	763	29.6	0.884	0.834	0.294	0.291
90	449	764	31.0	0.867		0.279	

**Table 4:** Mechanical properties and formability parameters of DC06 steel (material C).

Dir. [°]	R_p0.2_ [MPa]	R_m_ [MPa]	A_80_ [%]	*r* [-]	*r_m_* [-]	*n* [-]	*n_m_* [-]
0	157	301	46.9	2.224		0.267	
45	159	302	46.0	1.552	1.812	0.256	0.261
90	148	298	49.8	1.921		0.265	

E—Young’s modulus, R_p0_._2_—yield stress at 0.2% elongation, R_m_—ultimate tensile stress, A_80_—total elongation, *r*—plastic strain ratio, *n*—strain hardening exponent, *n_m_*—average value of strain hardening exponent, *r_m_*—average value of plastic strain ratio.

**Table 5:** Hollomon hardening model parameters.

Material	*K* (MPa)	*n* (-)
HX420	791	0.144
RAK 40/70	1488	0.291
DC06	551	0.261

**Table 6:** Hill48 and Barlat91 parameters.

Material	*r*_0_ (-)	*r*_45_ (-)	*r*_90_ (-)	*σ*_0_ (MPa)	*σ*_45_ (MPa)	*σ*_90_ (MPa)
HX420	0.790	1.096	0.599	468	465	454
RAK 40/70	0.702	0.844	0.867	435	443	449
DC06	2.224	1.552	1.921	157	159	148

*r*_0_—plastic strain ratio in 0° to the rolling direction, *r*_45_—plastic strain ratio in 45° to the rolling direction, *r*_90_—plastic strain ratio in 90° to the rolling direction, *σ*_0_—yield stress in 0° to the rolling direction, *σ*_45_—yield stress in 45° to the rolling direction and *σ*_90_—yield stress in 90° to the rolling direction.

**Table 7:** Data obtained from simulation and deviation of predicted springback angle from the measured springback value for material A.

Yield Criterion	Element Size [mm]	Effective Plastic Strain [-]	Equivalent Stress [MPa]	Predicted Springback Angle [°]	Springback Angle from Experiment [°]	Deviation of Springback Prediction [°]
Hill48	0.34	0.12	282.44	9.85	45.60	35.75
	0.17	0.15	313.99	13.64		31.96
	0.085	0.17	324.88	14.11		31.49
Barlat91	0.34	0.12	282.44	9.88	45.60	35.72
	0.17	0.15	313.99	13.53		32.07
	0.085	0.17	324.88	14.15		31.45

**Table 8:** Data obtained from simulation and deviation of predicted springback angle from the measured springback value for material B.

Yield Criterion	Element Size [mm]	Effective Plastic Strain [-]	Equivalent Stress [MPa]	Predicted Springback Angle [°]	Springback Angle From Experiment [°]	Deviation of Springback Prediction [°]
Hill48	0.37	0.21	366.53	15.25	50.20	34.95
	0.185	0.23	383.75	17.75		32.45
	0.0925	0.23	388.3	17.90		32.30
Barlat91	0.37	0.21	366.48	15.24	50.20	34.96
	0.185	0.23	383.75	17.74		32.46
	0.0925	0.23	388.31	18.56		31.64

**Table 9:** Data obtained from simulation and deviation of predicted springback angle from the measured springback value for material C.

Yield Criterion	Element Size [mm]	Effective Plastic Strain [-]	Equivalent Stress [MPa]	Predicted Springback Angle [°]	Springback Angle from Experiment [°]	Deviation of Springback Prediction [°]
Hill48	0.42	0.05	103.15	11.27	14.60	3.33
	0.21	0.06	132.26	11.56		3.04
	0.105	0.08	153.29	11.90		2.70
Barlat91	0.42	0.05	123.48	8.60	14.60	6.00
	0.21	0.06	144.29	8.68		5.92
	0.105	0.10	164.43	8.85		5.75

## Data Availability

The original contribution presented in the study are included in the article, and further inquiries can be directed to the corresponding author.
